# The haplotype-resolved T2T genome of teinturier cultivar Yan73 reveals the genetic basis of anthocyanin biosynthesis in grapes

**DOI:** 10.1093/hr/uhad205

**Published:** 2023-10-13

**Authors:** Kekun Zhang, Mengrui Du, Hongyan Zhang, Xiaoqian Zhang, Shuo Cao, Xu Wang, Wenrui Wang, Xueqiang Guan, Penghui Zhou, Jin Li, Wenguang Jiang, Meiling Tang, Qiuling Zheng, Muming Cao, Yongfeng Zhou, Keqin Chen, Zhongjie Liu, Yulin Fang

**Affiliations:** College of Enology, Heyang Viti-Viniculture Station, Ningxia Helan Mountain's East Foothill Wine Experiment and Demonstration Station, Northwest A&F University, Yangling 712100, China; National Key Laboratory of Tropical Crop Breeding, Shenzhen Branch, Guangdong Laboratory of Lingnan Modern Agriculture, Key Laboratory of Synthetic Biology, Ministry of Agriculture and Rural Affairs, Agricultural Genomics Institute at Shenzhen, Chinese Academy of Agricultural Sciences, Shenzhen, 518120, China; National Key Laboratory of Tropical Crop Breeding, Shenzhen Branch, Guangdong Laboratory of Lingnan Modern Agriculture, Key Laboratory of Synthetic Biology, Ministry of Agriculture and Rural Affairs, Agricultural Genomics Institute at Shenzhen, Chinese Academy of Agricultural Sciences, Shenzhen, 518120, China; College of Agriculture, Shanxi Agricultural University, Taigu 030801, China; College of Enology, Heyang Viti-Viniculture Station, Ningxia Helan Mountain's East Foothill Wine Experiment and Demonstration Station, Northwest A&F University, Yangling 712100, China; College of Enology, Heyang Viti-Viniculture Station, Ningxia Helan Mountain's East Foothill Wine Experiment and Demonstration Station, Northwest A&F University, Yangling 712100, China; National Key Laboratory of Tropical Crop Breeding, Shenzhen Branch, Guangdong Laboratory of Lingnan Modern Agriculture, Key Laboratory of Synthetic Biology, Ministry of Agriculture and Rural Affairs, Agricultural Genomics Institute at Shenzhen, Chinese Academy of Agricultural Sciences, Shenzhen, 518120, China; National Key Laboratory of Tropical Crop Breeding, Shenzhen Branch, Guangdong Laboratory of Lingnan Modern Agriculture, Key Laboratory of Synthetic Biology, Ministry of Agriculture and Rural Affairs, Agricultural Genomics Institute at Shenzhen, Chinese Academy of Agricultural Sciences, Shenzhen, 518120, China; College of Enology, Heyang Viti-Viniculture Station, Ningxia Helan Mountain's East Foothill Wine Experiment and Demonstration Station, Northwest A&F University, Yangling 712100, China; Shandong Grape Research Institute, Shanda South Road, Jinan 250199, China; Shandong Technology Innovation Center of Wine Grape and Wine, COFCO Great Wall Wine (Penglai) Co., Ltd., Yantai 265600, China; Shandong Technology Innovation Center of Wine Grape and Wine, COFCO Great Wall Wine (Penglai) Co., Ltd., Yantai 265600, China; Yantai Changyu Group Co., Ltd., Yantai 264001, China; Yantai Academy of Agricultural Sciences, Gangcheng West Street, Yantai 264000, China; Yantai Academy of Agricultural Sciences, Gangcheng West Street, Yantai 264000, China; Viticulture and Wine Research Institute, Guangxi Academy of Agricultural Sciences, Nanning 530007, China; National Key Laboratory of Tropical Crop Breeding, Shenzhen Branch, Guangdong Laboratory of Lingnan Modern Agriculture, Key Laboratory of Synthetic Biology, Ministry of Agriculture and Rural Affairs, Agricultural Genomics Institute at Shenzhen, Chinese Academy of Agricultural Sciences, Shenzhen, 518120, China; National Key Laboratory of Tropical Crop Breeding, Tropical Crops Genetic Resources Institute, Chinese Academy of Tropical Agricultural Sciences, Haikou 570100, China; College of Enology, Heyang Viti-Viniculture Station, Ningxia Helan Mountain's East Foothill Wine Experiment and Demonstration Station, Northwest A&F University, Yangling 712100, China; National Key Laboratory of Tropical Crop Breeding, Shenzhen Branch, Guangdong Laboratory of Lingnan Modern Agriculture, Key Laboratory of Synthetic Biology, Ministry of Agriculture and Rural Affairs, Agricultural Genomics Institute at Shenzhen, Chinese Academy of Agricultural Sciences, Shenzhen, 518120, China; College of Enology, Heyang Viti-Viniculture Station, Ningxia Helan Mountain's East Foothill Wine Experiment and Demonstration Station, Northwest A&F University, Yangling 712100, China

## Abstract

Teinturier grapes are characterized by the typical accumulation of anthocyanins in grape skin, flesh, and vegetative tissues, endowing them with high utility value in red wine blending and nutrient-enriched foods developing. However, due to the lack of genome information, the mechanism involved in regulating teinturier grape coloring has not yet been elucidated and their genetic utilization research is still insufficient. Here, the cultivar ‘Yan73’ was used for assembling the telomere-to-telomere (T2T) genome of teinturier grapes by combining the High Fidelity (HiFi)， Hi-C and ultralong Oxford Nanopore Technologies (ONT) reads. Two haplotype genomes were assembled, at the sizes of 501.68 Mb and 493.38 Mb, respectively. In the haplotype 1 genome, the transposable elements (TEs) contained 32.77% of long terminal repeats (LTRs), while in the haplotype 2 genome, 31.53% of LTRs were detected in TEs. Furthermore, obvious inversions were identified in chromosome 18 between the two haplotypes. Transcriptome profiling suggested that the gene expression patterns in ‘Cabernet Sauvignon’ and ‘Yan73’ were diverse depending on tissues, developmental stages, and varieties. The transcription program of genes in the anthocyanins biosynthesis pathway between the two cultivars exhibited high similarity in different tissues and developmental stages, whereas the expression levels of numerous genes showed significant differences. Compared with other genes, the expression levels of *VvMYBA1* and *VvUFGT4* in all samples, *VvCHS2* except in young shoots and *VvPAL9* except in the E-L23 stage of ‘Yan73’ were higher than those of ‘Cabernet Sauvignon’. Further sequence alignments revealed potential variant gene loci and structure variations of anthocyanins biosynthesis related genes and a 816 bp sequence insertion was found in the promoter of *VvMYBA1* of ‘Yan73’ haplotype 2 genome. The ‘Yan73’ T2T genome assembly and comparative analysis provided valuable foundations for further revealing the coloring mechanism of teinturier grapes and the genetic improvement of grape coloring traits.

## Introduction

Grapes are widely cultivated throughout the world, the berries of which can be consumed as table fruits and used to make wines or raisins. The coloring characteristics and anthocyanins accumulation are key traits of concern in grape genetic breeding and cultivation regulation. Anthocyanins are one of the most important secondary metabolites in plants, not only affecting the colors of plants but also contributing to their nutritional quality. Anthocyanins are synthesized through phenylalanine metabolism and flavonoid metabolism in grapes, catalyzed by a series of multi-enzyme complexes attached to the cell membrane and endoplasmic reticulum membrane [1].

Anthocyanins biosynthesis initially takes place in the cytoplasm of grape berry epidermal cells, and then they are stored in vacuoles through vesicle transport [2] and membrane transporters [3]. The biosynthesis of anthocyanins in grape berries can be regulated by multiple transcription factors. *VvMYBA1* and *VvMYBA2* on chromosome 2 played a major role in regulating the coloration of the grape pericarp, and they formed a highly linked gene cluster region regulating the production of anthocyanins by altering the expression of the glycosyltransferases VvUFGT, acyltransferase Vv3AT and vacuolar membrane H^+^ pump enzyme VvVHP1/2 [4–8]. In addition to VvMYBA1/2, other MYB members, such as VvMYB5a/b [9, 10] and VvMYBA6/7 [11], and other types of regulatory factors, such as VvMYC1, VvMYCA1, miRNA828, and miRNA858 [12–14]*,* can also function by regulating the expression of anthocyanins biosynthesis related genes.

Generally, anthocyanins accumulate only in grape skin but not in the flesh. There is a type of grape variety called teinturier cultivars, in which anthocyanins can accumulate not only in grape skin but also in the flesh and vegetative tissues. Teinturier grapes are a unique germplasm that can be used for red wine blending and the development of nutrient-rich foods. Previous studies identified that the malvidin and peonidin derivatives were the dominant components in the skin and flesh of some teinturier varieties, respectively, and the total anthocyanins content was higher in the skin [15–18]. In the vegetative tissues of teinturier grapes, 3’-OH, acetylated, and methoxylated anthocyanins were the main forms of anthocyanin accumulation [19]. Furthermore, the accumulation patterns of anthocyanins in the skin and flesh of different teinturier grapes might be divergent. ‘Gamay de Bouze’ and ‘Gamay Fréaux’ were the two red flesh variants of ‘Gamay’ with different beginning time of flesh coloring. ‘Gamay de Bouze’ accumulated anthocyanins from the veraison stage, while ‘Gamay Fréaux’ accumulated anthocyanins from the fruit setting stage [18].

The regulation of grape flesh coloring has been studied but the mechanism has not yet been elucidated. VvMYBA1 might regulate the anthocyanins biosynthesis in flesh by activating the promoters of *VvCHI3*, *VvOMT*, and *VvGST4*, while VvMYBC2-L1 could compete with MYB transcription factors for the binding site of bHLH, or negatively regulate grape flesh coloring by inhibiting the expression of *VvOMT* and *VvGST4* [20]. Meanwhile, *VvMYBA1, VvMYBA5, VvMYBA6,* and *VvMYBA7* might be jointly involved in the regulation of anthocyanin synthesis in vegetative tissues [19]. Röckel *et al.* analysed the promoters of *VvMYBA1* in three clones of teinturier grape ‘Rubitraube’ with different color intensities and found that the number of a 408 bp repetitive DNA sequence (GCE) in the promoter region was associated with the content of anthocyanins and the expression level of anthocyanins biosynthesis related genes [21]. However, previous studies have only focused on the alignment of a few functional genes or mining key genes solely relying on the transcriptome profiles. The differences between teinturier grapes and other varieties at the whole genome level, as well as the potential regulatory variant loci still need further investigation.

A high-quality genome is indispensable for important traits study and key genes discovery. Benefiting from the development of sequencing technology, the genomes of several wine varieties and rootstock varieties have been assembled successively (http://www.grapegenomics.com/) and the telomere-to-telomere gap-free grape reference genome have also been released [22], providing technical support for the excavation of key regulatory gene loci. The grape *Vitis vinifera* L. cv. ‘Yan73’ is the main teinturier grape variety planted in China, cultivated by Yantai Changyu Pioneer Wine Company Limited in 1966 and is the hybrid offspring of *V. vinifera* cv. Alicante Bouschet and *V. vinifera* cv. Muscat Hamburg. In this study, the T2T genome of ‘Yan73’ grape was constructed by the HiFi sequencing, Hi-C technology and ONT sequencing. Through comparative analysis of the genome and transcriptome, potential variant regulatory loci were discovered, providing the genetic basis for further revealing the regulatory mechanism of teinturier grape coloring.

## Results

### The T2T genome for ‘Yan73’

The ‘Yan73’ sequencing on the PacBio Sequel II platform generated a total of 29.41 Gb HiFi reads and two haplotypes were finally assembled (Table 1). The heterozygosity of the genome was 1.35% as calculated by the k-mer metric (Fig. S1, see online supplementary material). The contig N50 reached 26.53 Mb in haplotype 1 (hap1) and 25.20 Mb in haplotype 2 (hap2). The whole genome size was 501.68 Mb in hap1 and 493.38 Mb in hap2, respectively (Table 1).

**Table 1:** Comparison of genomic features between ‘Yan73’ and PN_T2T

**Items**	**Hap1**	**Hap2**	**PN_T2T**
Total sequence length (bp)	501 678 942	493 383 697	494 873 210
Number of chromosomes	19	19	19
Contig N50 (bp)	26 525 800	25 195 561	25 934 928
Maximum length (bp)	38 015 129	38 312 436	36 684 271
Number of gaps	0	4	0
Bases masked (bp)	340 392 442	332 882 486	328 929 883
Retroelements (bp)	189 198 000	180 188 509	241 027 616
LTR elements(bp)	164 401 990	155 587 657	235 245 099
Telomeres annotated	36/38	36/38	36/38
Number of TEs	453 619	445 431	935 783
BUSCO (%)	98.40	98.10	98.50

**Figure 1:**
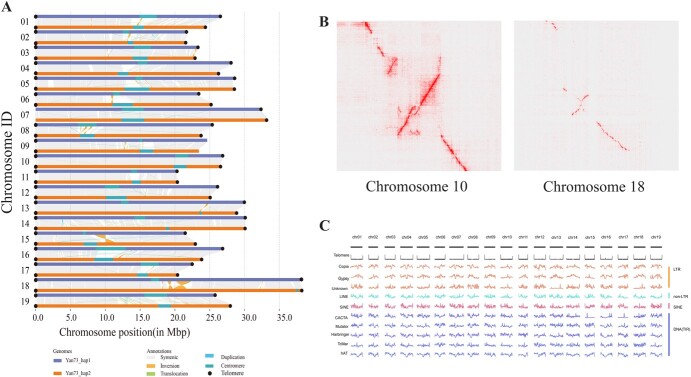
T2T assembly of the ‘Yan73’ genome. **A** Syntenic analysis between haplotype 1 and haplotype 2 genomes. The distribution of telomeres and centromeres was marked on the chromosomes. **B** Inversions of chromosome 10 and chromosome 18 visualized in the Hi-C map. **C** The chromosomal distribution of different types of transposable elements (TEs) annotated in the ‘Yan73’ genome.

Syntenic analysis between haplotype 1 and haplotype 2 of the ‘Yan73’ genome was carried out, and a collinearity map between the two haplotypes was constructed using Plotsr software (Fig. 1A). The results indicated that there were different types and quantities of chromosomal variations on each chromosome. Except for chromosomes 17, translocations were found on all other chromosomes. Duplications were present on most chromosomes, with a higher number on chromosomes 2, 14, 15, 16, and 19. Except for chromosomes 4, 7, 10, and 11, inversions were detected on all other chromosomes. Furthermore, the distribution of telomeres and centromeres on chromosomes was also marked. Two telomeres were identified on most chromosomes, except for the chromosome 7 and 9 with one telomere, respectively. To present chromosomal variations more clearly, a complete Hi-C map was generated based on the 3D-DNA data (Fig. S2, see online supplementary material). The pseudo-chromosome/chromosome was magnified on the Hi-C map, and obvious inversions were found on chromosome 10 and chromosome 18, respectively (Fig. 1B). Additionally, MUMmer (v.4.0.0rc1) was used to further verify the variation. However, when compared with the reference genome, only the variation in chromosome 18 was confirmed to be a true inversion (Fig. S3).

To ensure the quality of the genome assembly, the assembled ‘Yan73’ grape genome was compared with the first T2T complete grape genome PN40024 (PN_T2T) (Fig. S4, see online supplementary material) and the results suggested that there was a strong linear relationship between them. The genome completeness evaluated using BUSCO indicated that over 98.0% of the core conserved plant genes were exhibited in the ‘Yan73’ genome. Therefore, the high-quality genomes were successfully assembled (Fig. S5, see online supplementary material).

### Genome annotation and repeat sequence identification

RepeatMask was used to identify the repetitive sequences of each genome and classify all identified duplicate elements into different categories (Fig. 1C). A total of 72 telomere regions were identified in the ‘Yan73’ genome, except for the tip of chromosome 7 and the end of chromosome 9 (Fig. 1C). The specific locations of centromeres on the chromosome were also identified through the quarTeT toolkit (Table S1). In hap 1 of ‘Yan73’, 32.77% of transposable elements (TEs) were annotated as long terminal repeats (LTRs), among which Copia and Gypsy accounted for 14.38% and 16.02%, respectively, while 11.69% of TEs were annotated as DNA terminal inverted repeats (TIRs). In hap2 genome, LTRs accounted for 31.53% of the TEs, among which Copia and Gypsy accounted for 14.37% and 14.89%, respectively, while DNA TIRs accounted for 11.58%.

**Figure 2:**
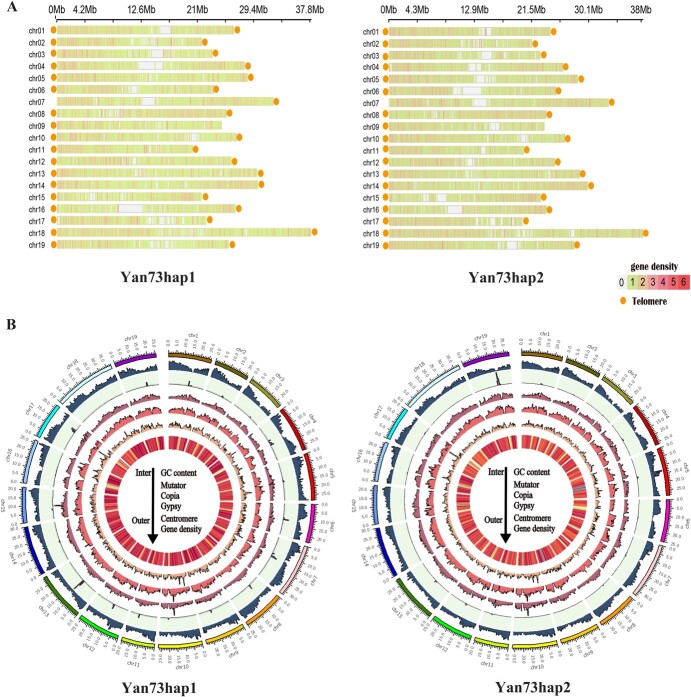
‘Yan73’ genome annotation. **A** Gene density and the distribution of telomeres in two haplotypes of the ‘Yan73’ genome. The ordinate is the chromosome number, and the abscissa is the position on the chromosome. 0–6 indicates the color degree of gene density. The orange points represent the telomeres on each chromosome. **B** Genome characteristics of ‘Yan73’. GC content and density of the mutators, Copia LTRs, Gypsy LTRs, centromere regions, and total genes were distributed from inside to outside the circle, respectively.

According to the genome annotation information of deleted duplicate prediction obtained from the final annotation, bedtools (v.2.30.0) was used to obtain the gene density file (Fig. 2A). In general, except for chromosomes 8, 15, and 16, the gene density was higher in the anterior region of the chromosome. The GC content, TE type and gene density of 19 chromosomes were also visualized by the Circos (Fig. 2B). After genome evaluation and annotation, the genetic characteristics of the ‘Yan73’ genome were calculated and compared with those of PN_T2T (Table 1). The contig N50 of Hap1 and Hap2 was 26 525 800 bp and 25 195 561 bp, respectively, close to the 25 934 928 bp of PN_T2T.

### Multiple genome alignment

**Figure 3:**
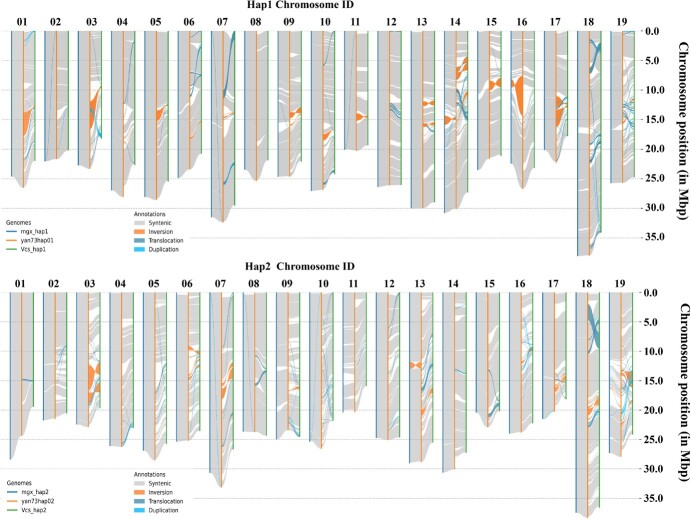
Multiple genome alignment of ‘Yan73’, ‘Muscat Hamburg’, and ‘Cabernet Sauvignon’. The syntenic region and different types of variation including inversion, translocation, and duplication were marked. Mgx, ‘Muscat Hamburg’; Vcs, ‘Cabernet Sauvignon’.

To reveal the genomic differences determining the formation of variety characteristics, multiple genome alignment including ‘Yan73’, ‘Muscat Hamburg’ and ‘Cabernet Sauvignon’ was conducted (Fig. 3). The results indicated that the collinearity effect of ‘Yan73’ and ‘Muscat Hamburg’ was more obvious, corresponding to the genetic relationship between them. By comparing haplotypes of different varieties, there were many chromosomal variations deserving attention. On chromosome 3 of haplotype 1, there was a larger inversion between ‘Yan73’ and ‘Cabernet Sauvignon’, while on chromosome 16 of haplotype 2, there was a larger inversion between ‘Yan73’ and ‘Muscat Hamburg’, which could be further studied and verified. In addition, compared with ‘Cabernet Sauvignon’, there was a large translocation region on chromosome 18 of ‘Yan73’. The above differential regions or variation sites might have a significant impact on the formation of special traits of ‘Yan73’.

### Overall gene expression patterns in different tissues and developmental stages of ‘Yan73’ and ‘Cabernet Sauvignon’

**Figure 4:**
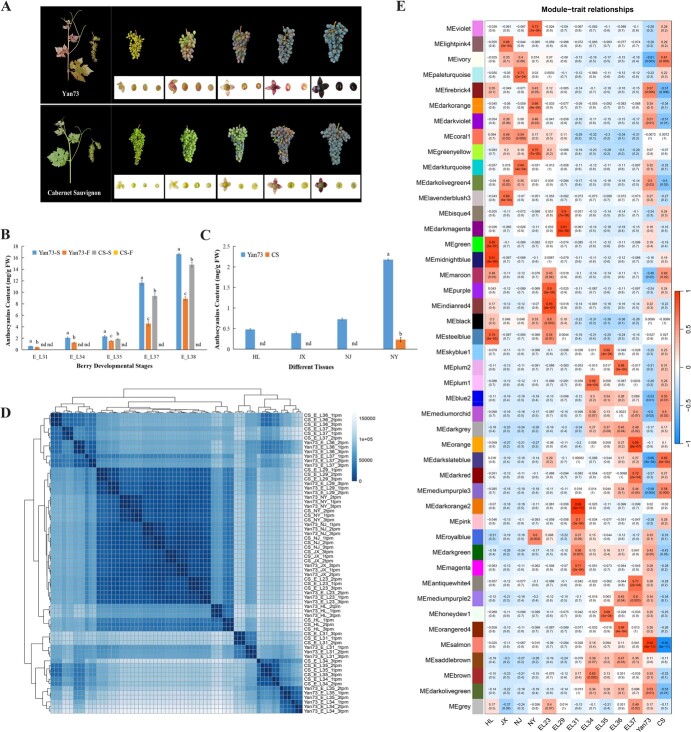
Gene expression patterns in different tissues and developmental stages of grape berries of ‘Yan73’ and ‘Cabernet Sauvignon’. **A** The coloring traits of different tissues of ‘Yan73’ and ‘Cabernet Sauvignon’. The berries were collected from stages of E-L31, E-L34, E-L35, E-L37, and E-L38, respectively. **B** Anthocyanins content in berries at different developmental stages. Yan73-S and Yan73-F indicates the skin and flesh of ‘Yan73’. CS-S and CS-F indicates the skin and flesh of ‘Cabernet Sauvignon’. The different letters indicate significant differences between ‘Yan73’ and ‘Cabernet Sauvignon’ at *P* < 0.05 (Duncan’s multiple comparison test). The same below. **C** Anthocyanins content of different tissues. HL, JX, NJ, and NY indicate inflorescences, tendrils, young shoots, and young leaves, respectively. The same below. **D** The cluster-heatmap of all samples from ‘Yan73’ and ‘Cabernet Sauvignon’ based on the RNA-seq data. **E** Heatmap of the correlation relationship between different modules and samples from ‘Yan73’ and ‘Cabernet Sauvignon’.

‘Cabernet Sauvignon’ is the most widely cultivated wine grape in the world. The black skin color, superior comprehensive quality and outstanding stress resistance make it become the typical representative of red wine grape varieties. To reveal the regulatory mechanism of teinturier grape coloring, ‘Cabernet Sauvignon’ was selected as a comparative partner of ‘Yan73’ for coloring traits analysis. Compared with the teinturier grape ‘Yan73’, the anthocyanins accumulation patterns of ‘Cabernet Sauvignon’ berries and vegetative tissues were different (Fig. 4A). The leaves, tendrils, young shoots, and inflorescences of ‘Yan73’ all appeared red during normal growth and development. At the fruit setting stage, the flesh and skin of the fruit were initially pigmented. The color intensity and the accumulation of anthocyanins in the flesh and skin steadily increased with berry development (Fig. 4A and B). Unlike ‘Yan73’, the young shoots, tendrils and inflorescences of ‘Cabernet Sauvignon’ remained green, while the young leaves were pigmented slightly red with the accumulation of a small amount of anthocyanins (Fig. 4A and C). During the ripening period, the skin of ‘Cabernet Sauvignon’ appeared purple black, and the flesh remained colorless.

To further clarify the mechanism of ‘Yan73’ vegetative tissues and flesh coloring, the shoots, leaves, tendrils, inflorescences, and berries at different developmental stages (E-L23, E-L29, E-L31, E-L34, E-L35, E-L36, and E-L37) of ‘Cabernet Sauvignon’ and ‘Yan73’ were selected for RNA sequencing and comparative analysis (Fig. 4D). The clustering heatmap of the transcriptome profiles showed that the gene expression patterns were significantly correlated with tissues, developmental stages and varieties. For berries in the E-L34, E-L35, E-L36, and E-L37 stages, the transcriptional differences caused by the developmental stage were higher than the effects caused by variety differences. For berries in stages of E-L29 and 31, tendrils, leaves, young shoots, and inflorescences, there was a high similarity in gene expression patterns between different varieties at the same developmental stage and tissues. The transcriptional differences between different developmental stages and tissues in these samples were greater than those caused by varieties. From the cluster heatmap, it can also be observed that there was a significant similarity between the leaves, young shoots, tendrils and berries at the E-L23 and E-L29 stages, which were significantly different from the samples at the flowering stage.

To further clarify the relationship between the expression patterns of different genes with varieties, tissues, and developmental stages, a weighted gene correlation network analysis (WGCNA) was conducted based on the 37 477 expressed genes from all samples and 45 co-expression modules were displayed in the figure with different colors (Fig. 4E). Different gene modules exhibited different correlation coefficients with varieties, tissues, and developmental stages.

Inflorescences, tendrils, leaves, and young shoots showed the highest positive correlations with the MEmidnightblue, MElavenderblush3, MEgreenyellow, and MEpaleturquoise modules, respectively. The genes in the MEsalmon module had the largest positive and negative correlations with ‘Yan73’ and ‘Cabernet Sauvignon’, respectively. From the perspective of gene expression mode, the gene expression level in this module was higher in all stages and tissues of ‘Yan73’ than that in’Cabernet Sauvignon’ (Fig. S6, see online supplementary material). Based on the GO database, the differentially expressed genes (DEGs) in this module were mainly enriched in metabolic processes related to DNA damage response and repair, as well as regulation of fatty acid biological process and lipid metabolic process, and regulation of response to osmotic stress (Fig. S7, see online supplementary material). To further clarify the differences in gene expression levels between ‘Yan73’ and ‘Cabernet Sauvignon’, 82 genes with significant differences in each developmental stage or tissue between ‘Yan73’ and ‘Cabernet Sauvignon’ were screened. The functions of these genes were diverse (Table S2, see online supplementary material), involved anthocyanin synthesis-, transport- and regulation-related genes, disease resistance proteins, and tissue growth-related genes. In addition, GO analysis of the DEGs between the same tissues of ‘Yan73’ and ‘Cabernet Sauvignon’ was also carried out (Figs S8–S11, see online supplementary material), and the different enriched metabolic processes in different tissues of the two cultivars were present.

### Differential expression patterns of genes related to the anthocyanins biosynthesis pathway

**Figure 5:**
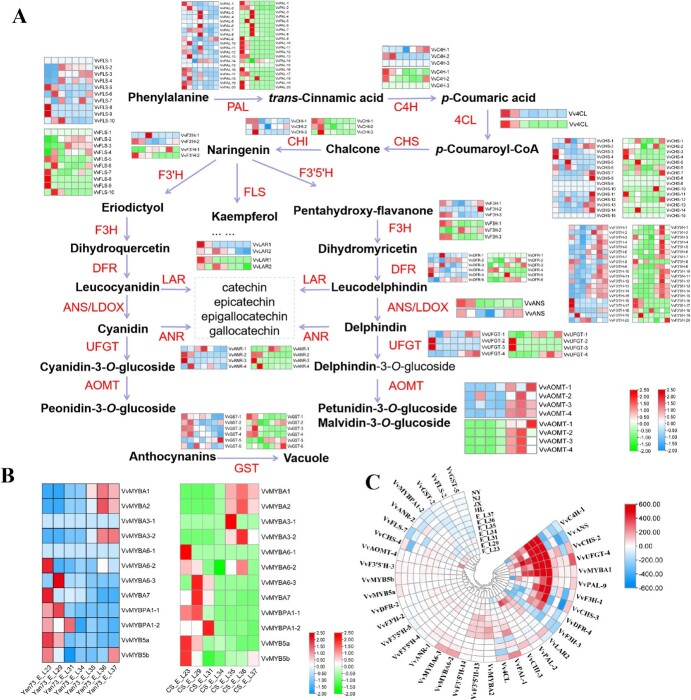
Comparative analysis of the expression levels of genes related to the anthocyanins biosynthesis pathway between ‘Yan73’ and ‘Cabernet Sauvignon’. **A** Comparison of gene expression patterns in the anthocyanins biosynthesis pathway of the whole berry at different developmental stages. The two heatmaps of each gene represent ‘Yan73’ (the former) and ‘Cabernet Sauvignon’ (the latter), respectively, and the horizontal axis of the heatmap represents different developmental stages in order of E-L23 to E-L37. **B** Comparison of gene expression patterns of MYB regulatory factors related to anthocyanins biosynthesis. **C** Cluster heatmap of the relative expression levels of 36 screened genes of ‘Yan73’ comparing with ‘Cabernet Sauvignon’.

To further explore the mechanism of anthocyanins accumulation differences, the expression levels of anthocyanins biosynthesis- and regulation-related genes in ‘Cabernet Sauvignon’ and ‘Yan73’ were compared and analysed. From E-L23 to E-L37, the expression of most of the anthocyanins biosynthesis and regulatory genes in the berries of ‘Cabernet Sauvignon’ and ‘Yan73’ showed similar developing trends (Fig. 5A and B), but the expression levels of numerous genes were significantly different between the two varieties (Fig. 5C).

During fruit development, the expression level of *VvPALs* generally presented a downward trend after the E-L31 stage, except for the *VvPAL9* and *VvPAL14* genes. The expression patterns of *VvUFGTs* and *VvAOMTs* showed a significant dependence on the developmental stage. *VvUFGT2* and *VvUFGT3* were mainly expressed during the full flowering period of E-L23, while *VvUFGT1* and *VvUFGT4* were mainly expressed in the later stage of fruit development. *VvAOMTs* were mainly expressed after the E-L34 stage, but there were differences in the expression trends of *VvAOMT1* and *VvOMT2* in the berries of ‘Cabernet Sauvignon’ and ‘Yan73’. Except for *VvGST6*, the expression of *VvGSTs* also showed similar dependence on development stages. The expression of MYB regulatory factors was also strongly correlated with different developmental stages. In the berries of the two varieties, *VvMYBA1* and *VvMYBA2* were mainly expressed after the E-L31 stage, while *VvMYBA6–2, VvMYBA6–3, VvMYBA7, VvMYBPA1–1, VvMYBPA1–2,* and *VvMYB5a* were mainly expressed in the early stages of development, and *VvMYB5b* was highly expressed at the E-L23 and E-L37 stages.

Comparative analysis was also conducted on the differences in the expression levels of anthocyanins biosynthesis and regulatory genes among different tissues (Fig. S12, Table S3, see online supplementary material), and there were differences in the tissue-specific expression trends of some genes. *VvPAL1* and *VvPAL2* were mainly expressed in leaves, while *VvPAL9, VvPAL10, VvPAL11, VvPAL12, VvPAL13, VvPAL14, VvPAL16, VvPAL17, VvPAL19,* and *VvPAL20* were mainly expressed in inflorescences. *VvC4H1* and *Vv4CL* were mainly expressed in young leaves. In ‘Yan73’, *VvCHS1, VvCHS2, VvCHS5, VvCHS6, VvCHS7, VvCHS11, VvCHS12, VvCHS13,* and *VvCHS14* were mainly expressed in inflorescences, while in ‘Cabernet Sauvignon’, *VvCHS2* to *VvCHS7*, and *VvCHS10* to *VvCHS14* were mainly expressed in young leaves. The tissue-specific expression trend of *VvUFGTs* was also similar in the two varieties. *VvUFGT1* and *VvUFGT3* were mainly expressed in inflorescences, *VvUFGT2* was expressed in tendrils, and *VvUFGT4* was mainly expressed in leaves. *VvAOMTs, VvGST1*, and *VvGST6* were also mainly expressed in leaves, while *VvGST2, VvGST3, VvGST4*, and *VvGST5* were highly expressed in inflorescences. *VvMYBA1* and *VvMYBA2* were also mainly expressed in inflorescences, while *VvMYBA6s, VvMYB5a,* and *VvMYB5b* were mainly expressed in young leaves.

To further reveal the differences in anthocyanins accumulation between the two varieties, a comparative analysis was conducted on the differences in the expression levels of anthocyanins biosynthesis- and regulation-related genes between the two varieties (Fig. S13, see online supplementary material). Based on the clustering analysis results and expression difference levels, 36 DEGs were selected and presented in Fig. 5C. At the fruit development stage of E-L35 to E-L37, the expression levels of *VvC4H-1, VvANS, VvCHS-2, VvUFGT-4, VvMYBA1, VvPAL-9, VvF3H-1, VvCHS-3*, and *VvDFR4* in ‘Yan73’ were significantly higher than those in ‘Cabernet Sauvignon’. Compared with other genes, *VvMYBA1* and *VvUFGT4* were the most noteworthy due to their higher transcription levels in all developmental stages and tissues of ‘Yan73’ than in those of ‘Cabernet Sauvignon’. Furthermore, the expression levels of *VvCHS2* (except for young shoots) and *VvPAL9* (except for the E-L23 stage) in ‘Yan73’ were also higher than those in ‘Cabernet Sauvignon’. In addition, the expression level of *VvGST2* was lower in all samples than that in ‘Cabernet Sauvignon’ among the genes down-regulated in ‘Yan73’.

### Candidate variant loci involved in the regulation of anthocyanins accumulation in ‘Yan73’

**Figure 6:**
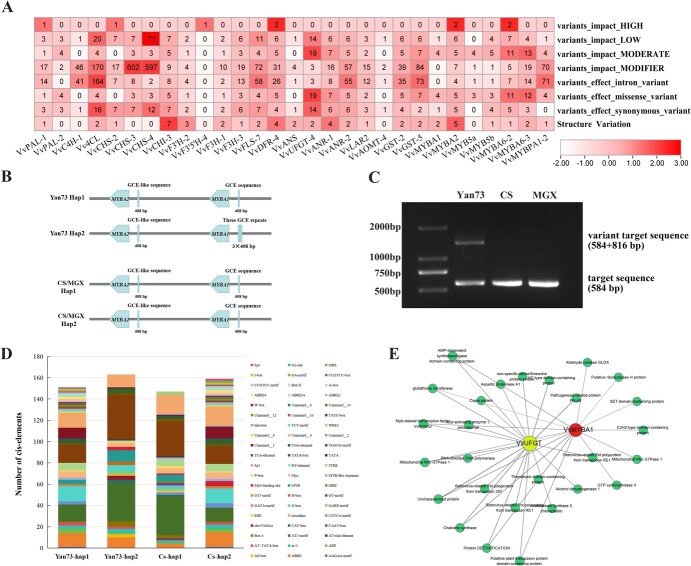
Identification and analysis of sequence variation of genes related to anthocyanins biosynthesis. **A** Variation types of genes related to anthocyanins biosynthesis. **B** Schematic diagram of *MYBA1* promoter regions in different haplotypes. CS, ‘Cabernet Sauvignon’; MGX, ‘Muscat Hamburg’. The same below. **C** PCR results for verifying the insertion variation in the promoter of *MYBA1* in ‘Yan73’ genome*.***D***Cis*-elements distributed in the promoter of *VvMYBA1* in ‘Yan73’ and ‘Cabernet Sauvignon’ genome. **E** The correlation network of *VvMYBA1* in the MEmediumpurple2 module.

Sequence variation may cause changes in gene expression levels and gene function [23, 24], leading to differences in anthocyanins biosynthesis pathways. To further explore the regulatory mechanism of anthocyanins accumulation in ‘Yan73’ flesh and vegetative tissues, the sequence differences in genes and promoters of the 32 screened genes were analysed and 29 variant genes were identified. SneEff was used for variant type annotation and analysis. The numbers of different types of variations, including the influence of sequence variation in exon regions of different genes (variants_impact_HIGH, variants_impact_LOW, variants_impact_MODERATE, variants_impact_ MODIFIER), missense and nonsynonymous mutations in exon regions (variants_effect_missense_variant and variants_effect_synonymous_variant), variation sites in intron regions (variants_effect_intron_variant), and structure variations were present in the heatmap (Fig. 6A; Table S4, see online supplementary material). The type of variants_impact_High indicated that the variants probably caused the protein truncation or loss of function, variants_impact_MODERATE represented the variant that might change protein effectiveness, and variants_impact_LOW represented that the variant was unlikely to change protein behavior. Variants_impact_MODIFIER indicated that the variants usually affected non-coding genes, but the impact was difficult to predict accurately.

There were the most variants_impact_LOW type-related mutation sites in the *VvCHS4* gene, with 71, and the maximum number of the variants_impact_MODERATE type in *VvUFGT4* with 19. The most variants_impact_MODIFIER type related mutations were found in *VvCHS3*, with 602, and the number of this type of variation in *VvMYBA2* was 7. The variation in gene sequence may affect the accumulation efficiency of anthocyanins by altering the function of enzyme proteins, but the effect of the above gene sequence variations still needs further analysis and verification. Structure variations (SVs) usually refer to >50 bp large-scale nucleotide sequence changes in the genome. There were numerous SVs detected in the promoters of regulatory genes *VvMYBA1, VvMYBA2, VvMYBA6–2, VvMYBA6–3,* and *VvMYBPA1–2.* The changes in the expression level of these genes may be related to the structure variations in the promoter.

Due to the significant difference in the expression level of *VvMYBA1* in different varieties and its importance in anthocyanins accumulation regulation, a comparative analysis of *VvMYBA1* promoter region was conducted. One previously reported GCE sequence (408 bp) [21] was found in the promoter of ‘Yan73’ hap1 genome, while three tandem GCE repeats were found in the hap2 genome. By aligning the GCE sequence across the entire genome of ‘Yan73’, ‘Cabernet Sauvignon’, and ‘Muscat Hamburg’, the two GCE repeats insertion were only detected in the chromosome 2 (16 017 480–16 018 295 bp) of ‘Yan73’ hap2 genome (Fig. 6B; Table S5, see online supplementary material), so a target sequence (584 bp) covering the insertion site was amplified to verify the accuracy of genome sequencing. The results of PCR and Sanger sequencing confirmed the 816 bp sequence insertion in one haplotype genome of ‘Yan73’ (Fig. 6C; Table S6, see online supplementary material). Moreover, the type and distribution of *cis*-elements in the promoter of *VvMYBA1* of ‘Yan73’ and ‘Cabernet Sauvignon’ were also analysed (Fig. 6D). The types of *cis*-elements in the promoter regions of ‘Yan73’ and ‘Cabernet Sauvignon’ were similar. The number of CAAT-box and TATA-box in ‘Yan73’ haplotype 2 and Cabernet Sauvignon haplotype 1 genome was higher than that in other haplotypes. Due to the insertion of GCE repeats, the number of MYB binding elements in the promoter of *VvMYBA1* of ‘Yan73’ haplotype 2 genome was significantly higher than that in other haplotype genomes. However, whether the insertion of 816 bp sequence can cause continuous high expression of *VvMYBA1* and the involved regulatory mechanism still need further investigation.

To further explore the genes involved in the transcriptional regulation of *VvMYBA1*, the gene network involved in the MEmediumpurple2 module was analysed (Fig. 6E) and various types of functional genes or regulatory factors, such as MYBA2, CHS, alcohol dehydrogenase 1, and aldehyde oxidase GLOX, were identified to be closely related to the expression of *VvMYBA1* and *VvUFGT*. These factors may coordinate with each other and participate in the regulation of anthocyanins accumulation in ‘Yan73’.

## Discussion

Genomic sequencing and assembly are important foundations for discovering key genes and clarifying evolutionary relationships between different species. However, due to the presence of telomeres, centromeres, and ribosomal DNA (rDNA) regions with highly repetitive sequences clustered across the genome [25, 26] and sequencing technology limitations, there are many missing regions in the early stage of genome assembly. Since the first complete human X chromosome was published in 2020 [27], T2T assembly has become a research focus. T2T assembly technology was based on the third-generation sequencing platform technology, integrating PacBio high-fidelity long reads (HiFi), ultralong ONT, and Hi-C data sequencing and analysis technology. At present, the technique of telomere-to-telomere and gap-free reference genome assembly has been used in kiwifruit (*Actinidia chinensis*), watermelon (*Citrullus lanatus*), rice (*Oryza sativa* xian/indica) and Myrtaceae (*Rhodomyrtus tomentosa*) [28–31].

In grapes, the genome assembly has been completed for many cultivars, such as ‘Cabernet Sauvignon’ [32], ‘Chardonnay’ [33], ‘Black Corinth’, and ‘Merlot’ [34]. Furthermore, the Cantu lab at UC Davis also constructed a web portal with genomic data and analysis tools for wild and cultivated grapevines [35]. The recent release of the T2T version of the grape reference genome has made up for the incomplete assembly of centromeres, telomeres, and repetitive regions [24]. A total of 37 534 genes were identified in the new version of PN40024, while 34 930 and 34 919 genes were identified in the ‘Yan73’ haplotype 1 and haplotype 2 genomes, respectively. It was also found that there was an obvious inversion in the ‘Yan73’ genome, and the occurrence of this structure variation may cause changes in gene function and differential expression of genes in this region. From the comparison of multiple genomes, ‘Yan73’ had a strong collinearity with ‘Muscat Hamburg’, which may be related to the genetic relationship between the two cultivars. The genome comparison of ‘Yan73’ with ‘Muscat Hamburg’ and ‘Cabernet Sauvignon’ also presented regions of inversion, translocation, and replication on chromosomes, which may change the function and expression patterns of involved genes, making ‘Yan73’ show variety specificity [36].

Hybrid grape breeding developed rapidly in response to complex and ever-changing climate change and the threat of pests and diseases in the 19th and 20th centuries, and multiple teinturier varieties were selected during this period. These teinturier varieties are currently widely distributed worldwide. Previous studies conducted to identify the relationships among numerous teinturier varieties that expanded across Europe in the early 20th century found that most of the teinturier varieties studied are related to the internationally known cultivars ‘Morrastrel Bouschet’, ‘Alicante Bouschet’, ‘Petit Bouschet’, and ‘Jacquez’ combining the ampelographic characterization of the leaves and the use of molecular markers [37]. In China, ‘Yan73’ was the main cultivated teinturier variety for increasing red wine color during winemaking, and it also exhibited high application value for developing nutrient-enriched foods. ‘Yan73’ differed from other teinturier varieties in the biosynthesis of volatile compounds. More C6 aldehydes in the free form and alcohols in the bound form were found in ‘Kolor’, but a higher content of terpenes was found in ‘Yan73’ [38]. The accumulation patterns of anthocyanins in ‘Yan73’ and ‘Gamay Fréaux’ were similar. Their flesh is colored from the beginning of the fruit setting period, and the leaves and other vegetative tissues can also be pigmented [20]. The transcriptome data suggested that the higher expression levels of *VvUFGT, VvMYBA1, VvCHS2,* and *VvPAL9* in the leaves, tendrils, and inflorescences of ‘Yan73’ might reduce the tissue specificity of anthocyanins accumulation, and make the leaves, tendrils, and flesh of ‘Yan73’ pigmented.

To reveal the coloring mechanism of teinturier grapes, considerable explorations have been conducted. It has been shown that the up-regulated expression of the structural genes *VvOMT, VvAM3, VvGST, VvF3’5’H,* and *VvLDOX,* the regulatory genes *VvMYBA1, VvMYBA6,* and *VvMYBA7,* as well as the negative regulatory factor *VvMYBC2-L1* might jointly participate in the regulation of fruit pulp and vegetative tissues coloring [21, 22]. In this study, the expression patterns of anthocyanins biosynthesis related genes in ‘Yan73’ and ‘Cabernet Sauvignon’ were similar, but the transcriptional levels of numerous genes in ‘Yan73’ were higher than that in ‘Cabernet Sauvignon’. The co-expression network modules of *VvMYBA1* also agreed that multiple genes were involved in the regulation of the flesh and vegetative tissues coloration.

VvMYBA1 is the key transcription factor that regulates grape coloring [4, 6, 39], and could function by regulating the expression of genes such as *VvUFGT* and *Vv3AT*. The significant difference in the expression level of *VvMYBA1* in this study also suggested that it played an important regulatory role in flesh and vegetative tissue coloring. However, the specific regulatory mechanism is still unclear. Sequence insertion and base mutations are important sources of color variation in grape berries. The insertion of the retrotransposon Gret1 in the promoter region of *VvMYBA1* in grape led to a change in the skin color from red to white [4], and a base mutation in the coding region of *VvMYBA2* also caused a color change in grape skin [8]. Muscadine grape skin mainly presents two color types: black/purple or bronze. The mutation of the conserved amino acid residue Pro171-to-Leu in GST led to it encoding an inactive protein due to modifications within the H-binding site, which made the grape skin become bronze without affecting the biosynthesis of anthocyanins [40]. In apple, the allelic rearrangement of five direct tandem repeats of a 23-bp sequence generated an autoregulatory locus, leading to the continuous high expression of *MdMYB10* and the accumulation of anthocyanins in the flesh [41]. In grapes, the number of a 408 bp GCE sequence in the *VvMYBA1* promoter region was related to the color intensity of the teinturier grapes ‘Rubitraube’ [23]. The more GCE repeats there were in the promoter region, the higher the expression level of *VvMYBA1* itself and the *VvUFGT* gene, and the higher the content of anthocyanins in the flesh and vegetative tissues. In white ‘Chardonnay’ grapes, overexpression of the *VvMYBA1*pr: *VvMYBA* construct resulted in red/purple pigmentation of the berries from the veraison stage, while continuous overexpression of *VvMYBA1* (35S: *VvMYBA*) led to pigmented skin, pulp, and other tissues [6]. Compared with the genome of ‘Cabernet Sauvignon’ and ‘Muscat Hamburg’, the insertion of two GCE repeats (816 bp) were detected in the promoter of *VvMYBA1* of ‘Yan73’, but it was unclear whether it could affect the expression of *VvMYBA1*.

In addition to *VvMYBA1* and *VvUFGT*, other genes also exhibited notable differences in the transcription patterns between ‘Yan73’ and ‘Cabernet Sauvignon’. The expression level of *VvMYBA2* in berries of ‘Yan73’ during EL-31 to EL-37 stage was higher than that in ‘Cabernet Sauvignon’, but did not show differences in other vegetative tissues, while the expression level of *VvMYBA6* was higher in vegetative tissues of ‘Yan73’, but did not show significant differences during fruit development. MYBA2 is usually involved in the regulation of anthocyanins biosynthesis in fruits [8], while MYBA6 usually functioned in vegetative tissues [11]. The expression difference indicated that the tissue-specific expression of these two genes in ‘Yan73’ were enhanced and the excessive accumulation of anthocyanins in ‘Yan73’ involved the synergistic effect of multiple factors. PAL is the main rate-limiting enzyme of phenylpropanoid metabolism, which determines the formation of trans-cinnamic acid [42], while CHS is the initial enzyme of flavonoid metabolism, which determines the biosynthesis of various types of flavonoid components [43]. The high expression of *VvPAL9* and *VvCHS2* in ‘Yan73’ might provide sufficient substrates for flavonoids metabolism, making an indispensable contribution to the high accumulation of anthocyanins in ‘Yan73’. Sequence variation analysis also identified SNPs and structure variations in gene sequence of *VvCHS2, VvMYBA2, VvMYBA6–2, VvMYBA6–3*, and their promoters, which may affect their transcription and function. Overall, the typical coloring traits of teinturier grape ‘Yan73’ were formed under the synergistic regulatory effect of multiple genes. The haplotype resolved T2T genome assembly and comparative analysis provide potential genetic variation sites for further elucidating the regulatory mechanism of anthocyanins ectopic accumulation.

## Conclusion

In this study, the T2T genome of the teinturier grape ‘Yan73’ was assembled, with 34 930 and 34 919 genes identified in the haplotype 1 and haplotype 2 genomes, respectively. In the haplotype 1 genome, 32.77% of LTRs were annotated in TEs, while 31.53% of LTRs were annotated in haplotype 2. Furthermore, an obvious inversion was found on chromosome 18 of the ‘Yan73’ genome. Comparative analysis of transcriptome profiles indicated that the whole gene expression patterns in ‘Cabernet Sauvignon’ and ‘Yan73’ were diverse depending on tissues, developmental stages, and varieties. For anthocyanins biosynthesis pathway, the expression levels of *VvMYBA1* and *VvUFGT4* in all samples, *VvCHS2* except in young shoots and *VvPAL9* except in E-L23 stage of ‘Yan73’ were higher than those of ‘Cabernet Sauvignon’. Further sequence alignments revealed potential variant gene loci and structure variations of anthocyanins biosynthesis related genes, which might participate in the regulation of the ectopic accumulation of anthocyanins. The T2T genome assembly of ‘Yan73’ and comparative analysis provide important foundations for revealing the coloring mechanism of teinturier grapes and the further genetic improvement of grape coloring traits.

## Materials and methods

### Plant sample collection and sequencing of the ‘Yan73’ genome

The ‘Yan73’ and ‘Cabernet Sauvignon’ grapes were planted in the vineyard of the College of Enology at Northwest A&F University. Young leaves and new shoots of ‘Yan73’ were prepared for genome sequencing. Meanwhile, different tissues of ‘Yan73’ and ‘Cabernet Sauvignon’ grapes, including inflorescences, young shoots, tendrils, young leaves, and berries were collected for RNA sequencing. The berries were collected from the E-L23, E-L29, E-L31, E-L34, E-L35, E-L36, E-L37, and E-L38 stages, according to the E-L system for identifying the grape growth stage [44]. The collected samples were quickly frozen in liquid nitrogen and then sequenced. In addition, the anthocyanins content of different tissues of ‘Yan73’ and ‘Cabernet Sauvignon’ was determined according to the methanol-HCl method [8].

Based on the third-generation sequencing platform, the PacBio High Fidelity Long Reading Segment (HiFi), Oxford Nanopore Technology (ONT), and Hi-C technology were comprehensively utilized for ‘Yan73’ grape genome sequencing. The DNeasy Plant Mini Kit (QIAGEN, Hilden, Germany) was used for genomic DNA and RNA isolation following the manufacturer’s instructions. Subsequently, the qualified DNA was segmented and selected using BluePippin. After end repair and the addition of an A tail, connectors were connected at both ends of the segment to prepare a DNA library, which was then sequenced using the PacBio Sequel II platform [22].

According to the previous method [45], the extracted DNA was used for the Hi-C library construction through the process of crosslinking, chromatin digestion with the four-cutter restriction enzyme MboI and marking of DNA ends, ligation and purification, shearing, and biotin pull down and then the Illumina HiSeq X Ten platform (San Diego, CA, USA) with 150PE mode was applied for the library sequencing.

The genomic DNA was also sequenced on the ONT platform. For the ultra-long Nanopore library, the Ligation sequencing 1D Kit (Catalog No. SQK-LSK109, Oxford Nanopore Technologies, Oxford, UK) was used for the processing of genomic DNA (>50 kb) selected with the SageHLS HMW library system (Sage Science) according to the manufacturer’s instructions. Then PromethION (Oxford Nanopore Technologies) was used for the DNA library sequencing.

### Genome assembly and completeness evaluation

The genome assembly of ‘Yan73’ was conducted using HiFi, Hi-C, and ONT sequencing data and the embryophyta_odb10 database of BUSCO (v.5.2.2) was used for the quality and integrity evaluation of the genome [46]. Preliminary assembly was performed on HiFi data at the contig level. Fastp (v.0.23.2) was used to splice and filter the raw Hi-C data for quality control [47], and then Hifiasm (v.0.15.5) was used to assemble haplotypes of reads, complete the typing of the diploid genome, and generate two high-quality haplotypes. The first step of chromosome mounting in genome assembly was performed using Juicer (v.1.6) and the size of each chromosome was calculated. With the gap free PN40024 as the reference genome, Ragtag (v.2.1.0) was applied to connect adjacent contigs, scaffold assembly and repair, and scaffold merging of the two haplotypes obtained in the initial assembly, and then the scaffold was located at the chromosome level. The merged_nodups.txt file generated by Juicer and the total assembly file generated by Ragtag were used as input files for 3D-DNA to correct the incorrect assembly. Finally, two haplotypes of the ‘Yan73’ genome containing 0 and 4 gaps were generated.

The collinearity between different haplotypes was analysed using Plotsr and the chromosomal structure variation was verified by MUMmer (v.4.0.0rc1). Before assembly, Jellyfish (v.2.3.0) was used to evaluate the genome size of the HiFi reads. The location of the centromere on the chromosome was detected by the Quartet toolkit.

### Genome annotation and repeat sequence recognition

The repeat sequence and gene structure of the ‘Yan73’ genome were annotated according to the genome wide annotation pipeline (https://github.com/unavailable-2374/Genome-Wide-Annotation-Pipeline). RepeatMask (v.4.1.2-p1) was used to identify the repetitive sequences by comparing the ‘Yan73’ genome with a library containing known transposable primitive family sequences. These repeats were masked to avoid interference with further analysis and to help compare and annotate new genomic sequences. In addition, Trimmomatic (v.0.39) was also applied for RNA-seq preprocessing to remove low-quality bases and improve the accuracy of subsequent analysis. In this annotation, 33 paired-end sequencing RNA-seq data were used to annotate the ‘Yan73’ genome using HISAT2 (v. 2.2.1) software. Maker was used to predict potential transcripts and protein-coding genes. The final results were merged into a GFF3 file containing predicted transcripts and protein-coding gene information for further analysis. When annotating, homologs effectively discovered and annotated new transcripts by comparing them with homologous sequences in the database. Then, the position and structure of protein-coding genes were predicted from the DNA sequence. Finally, combineGeneModels was applied to integrate the gene prediction results from different software and data sources and combine these individual prediction results into a GFF3 file containing more complete and accurate genome annotation information and deleting duplicate predictions. The gene density file was generated by using bedtools (v2.30.0) according to the annotation results, and the gene density size was displayed.

### Genomic comparison and potential variable sites identification

The whole genomes of ‘Yan73’, ‘Muscat Hamburg’, and ‘Cabernet Sauvignon’ were compared with Minimap2, and the results were input into Synteny and Rearrangement Identifier (SyRI) software to identify the collinear regions and structural rearrangements (inversion, translocation, and duplication). Plotsr was used to visualize the differences between various genome structures.

The MUMmer software was used to conduct collinear alignment between the ‘Yan73’ and ‘Cabernet Sauvignon’ genomes, with PN40024 as the reference genome. Then, SyRI software was applied to analyse the comparison results and identify the local variation of the selected gene sequence regions. The variation results were finally annotated using SnpEff software (5.0e).

To verify the insertion of 816 bp sequence in the promoter of *VvMYBA1*, genomic DNA was extracted from ‘Yan73’, ‘Cabernet Sauvignon’, and ‘Muscat Hamburg’ leaves using the cetyltrimethylammonium bromide (CTAB) method, and primers were designed for the amplification of target sequence covering the inserted site (Table S6, see online supplementary material).

### Transcriptome data processing

RNA extraction and sequencing were performed according to the previously used method [48, 49]. The extracted RNA qualified for the integrity was used to generate the double-stranded cDNA through the steps of enrichment, random interruption, reverse transcription amplification, and purification. Then, the cDNA was amplified for library constuction, and the Illumina Novaseq platform was used for sequencing. The FPKM (expected number of fragments per kilobase of transcript sequence per millions base pairs sequenced) of each gene was calculated based on the length of the gene and read numbers mapped to the gene. The DESeq2 R package (1.16.1) was used to analyse the DEGs between the two comparison groups. The DEG-enriched pathways were analysed in the GO databases using R-clusterprofiler. The heatmap for the transcription comparison of anthocyanins biosynthesis pathway was made using TBtools (v1.125).

WGCNA was used to clarify the correlation of gene modules with development stages, tissues, and cultivars. Based on previous methods [28]，the co-expression similarity coefficient between genes and the weight of different samples was generated using the TOMsimilarity module and PickSoftThreshold functions of the R software package. An absolute value higher than 0.8 and the *P* value lower than 0.001 were used as the thresholds to screen the gene modules that were significantly correlated with development stages, tissues, and cultivars. The network of co-expressed genes was visualized using the Cytoscape platform (https://cytoscape.org/).

## Supplementary Material

supple_uhad205

## Data Availability

All the raw sequencing data generated for this project have been deposited in the National Genomics Data Center (NGDC) Genome Sequence Archive (GSA) (https://ngdc.cncb.ac.cn/gsa/) with BioProject number PRJCA018686, and in the NCBI Sequence Read Archive under project number PRJNA1000119. The assembly and annotation as well as the sequences of centromeres and heterozygous regions have been deposited in Zenodo.
